# The mineralocorticoid receptor antagonist eplerenone reduces renal interstitial fibrosis after long-term cyclosporine treatment in rat: antagonizing cyclosporine nephrotoxicity

**DOI:** 10.1186/1471-2369-14-42

**Published:** 2013-02-20

**Authors:** Finn Thomsen Nielsen, Boye L Jensen, Pernille BL Hansen, Niels Marcussen, Peter Bie

**Affiliations:** 1 Department of Cardiovascular and Renal Research, Institute of Molecular Medicine, University of Southern Denmark, Winsløwparken 21, 3, Odense C, DK-5000, Denmark; 2 Department of Nephrology, State University Hospital, Copenhagen, Denmark; 3 Department of Pathology, Odense University Hospital, University of Southern Denmark, Odense, Denmark

**Keywords:** Aldosterone, Calcineurin, Hypertension, Nephrotoxicity, Renin

## Abstract

**Background:**

Chronic cyclosporine-(CsA)-mediated loss of kidney function is a major clinical problem in organ transplantation. We hypothesized that the mineralocorticoid receptor antagonist eplerenone (EPL) prevents chronic CsA-induced renal interstitial volume increase, tubule loss, and functional impairment in a rat model.

**Methods:**

Sprague–Dawley rats received CsA alone (15 mg/kg/d p.o.), CsA and EPL (approximately 100 mg/kg/day p.o.) or vehicle (control) for 12 weeks. At 11 weeks, chronic indwelling arterial and venous catheters were implanted for continuous measurements of arterial blood pressure (BP) and GFR (inulin clearance) in conscious, freely moving animals. Plasma was sampled for analysis and kidney tissue was fixed for quantitative stereological analyses.

**Results:**

Compared to controls, CsA-treatment reduced relative tubular volume (0.73±0.03 vs. 0.85±0.01, p<0.05) and increased relative interstitial volume (0.080±0.004 vs. 0.045±0.003, p<0.05); EPL attenuated these changes (0.82±0.02, p<0.05, and 0.060±0.006, p<0.05, respectively). CsA-treated rats had more sclerotic glomeruli and a higher degree of vascular depositions in arterioles; both were significantly reduced in CsA+EPL-treated animals. CsA increased BP and reduced body weight gain and GFR. In CsA+EPL rats, weight gain, GFR and BP at rest (daytime) were normalized; however, BP during activity (night) remained elevated. Plasma sodium and potassium concentrations, kidney-to-body weight ratios and CsA whole blood concentration were similar in CsA and CsA+EPL rats.

**Conclusions:**

It is concluded that in the chronic cyclosporine rat nephropathy model, EPL reduces renal tissue injury, hypofiltration, hypertension, and growth impairment. MR antagonists should be tested for their renoprotective potential in patients treated with calcineurin inhibitors.

## Background

The calcineurin inhibitor cyclosporine A (CsA) is a powerful macrolide immunosuppressive agent widely used after organ transplantation. The calcineurin inhibitors exert major nephrotoxic effects that involve acute vasoconstriction related to afferent glomerular arterioles as well as a pro-fibrotic effect in the chronic phase [1,2]. Many treatment options have been examined with the purpose of countering these adverse effects, but until recently there have been no single pharmacological approach to prevent the gradual decline in renal function and the progressive renal fibrosis occurring during CsA treatment. There is solid evidence to indicate that aldosterone aggravates the CsA-induced nephrotoxicity [3-8] and consequently, that mineralocorticoid receptor (MR) blockade might have a preventive effect. In short-term animal studies (up to 21 days) the MR-antagonist spironolactone slowed the progression of renal dysfunction and reduced the morphological changes seen after CsA treatment in rats [3-7]. The more selective MR antagonist eplerenone (EPL) antagonized the deterioration of renal function and blood pressure (BP) increase occurring in the early stage (21 days of treatment) of CsA-treated rats [8]. With respect to adverse effects, EPL has a clinical profile superior to that of spironolactone [9]. Preventing the CsA-mediated renal fibrosis and loss of nephrons during chronic treatment is a major clinical challenge. However, in animal model studies appropriate quantitative techniques have been applied only for periods of 2–3 weeks. The present study was undertaken to test the hypothesis that the selective MR-antagonist EPL protects against renal epithelial cell loss and interstitial fibrosis in a long-term model (12 weeks) of CsA nephropathy. To assess the tissue volume fraction occupied by interstitium and tubules, a quantitative unbiased stereological method was applied to analyse fixed kidney sections from the CsA-induced rat nephropathy model. Arterial and venous catheters were implanted for continuous recordings of BP, determination of glomerular filtration rate GFR, and blood sampling in conscious, unstressed animals. Components of the renin-angiotensin system, CsA and, electrolytes were measured in plasma.

This study reports results of MR-inhibition in CsA-treated rats in which renal fibrosis, interstitial expansion, and loss of tubular mass were reduced, renal function preserved, and BP lowered in a 12 week model.

## Methods

### Experimental animals

Inbred, male Sprague–Dawley rats (Mol:SPRD) from Harlan Scandinavia (Harlan, Alleroed, Denmark) initially weighing 180–240 g were used. The rats had free access to tap water and a wet mash standard non-salt-reduced diet (Altromin® Standard 1320 with 0.2% sodium, Lage, Germany) and were housed in air-conditioned rooms at a 12h light and 12h dark (LD 12:12) cycle. The investigation was performed according to “Guide for the Care and Use of Laboratory Animal” published by the US National Institute of Health, and the experimental protocol was approved by the Danish Animal Experiments Inspectorate (j.no. 2007/561-1389).

### Drug preparation and treatment protocol

The rats were treated by a daily gavage with CsA (Sandimmune Neoral®, Novartis Pharma AG, Basel, Switzerland) for the CsA (n=11) and CsA+EPL (n=11) groups or vehicle (castor oil) only (n=11) for 12 weeks. The CsA dose was 15 mg/kg/d; the doses were adjusted in steps with the weight increase of the individual rat. For administration of EPL, tablets of 50 mg (Inspra®, Pharmacia Ltd., Northumberland, England) were crushed and added to the diet: 1.2 mg/g dry food approximating a daily dose of 100 mg/kg body weight [10]. The non-EPL treatment groups received a similar diet without EPL.

During the treatment period, six rats were excluded due to aspiration after CsA-administration. Two rats in the CsA-alone treatment group were excluded due to general weakness and dehydration; both of them showed signs of pneumonia at autopsy.

### Catheters in artery and vein

Chronic indwelling catheters were placed in the femoral vessels for arterial blood sampling, measurements of arterial BP, and intravenous infusions [11]. Animals were anaesthetised as described previously [8]. Following the operation the catheters were attached to a swivel enabling the rats to move freely irrespective of BP measurements, arterial blood sampling, and venous infusions.

### Blood pressure measurements and clearance studies

Two days after surgery, the arterial catheter was connected to a BP transducer linked to an amplifier (BLPR and BP1, World Precision Instruments, Hertfordshire, United Kingdom) and a computer running custom designed soft-ware (LabVIEW Real-Time®, version 7 Express, National Instruments^TM^, Dublin, Ireland) for continuous recording of BP and heart rate. BP was measured on-line during 96 hours. The venous catheter was connected to an infusion pump delivering inulin (Polyfructosan S, Laevosan®, Petrone Group, Napoli, Italy) 25% w/vol at a rate of 4 μl/kg/min. After four days it was assumed that steady state was obtained, i.e., the rate of excretion was constant and equal to the rate of infusion. Therefore, inulin clearance was calculated as infusion rate divided by plasma concentration. Arterial blood samples were centrifuged immediately and plasma separated and stored at -80°C. Separate whole blood samples were drawn for analysis of CsA concentrations.

### Analysis

Whole blood CsA concentrations (trough levels) were measured after 12 weeks of treatment in samples collected 22–26 hours after the last administration of CsA. Analyses were performed with a radioimmunoassay kit (TDx/TDxFLx; Abbott Laboratories, Abbott Park, IL, USA).

Inulin concentrations in plasma were analysed by the diphenylamine method [12] modified for microanalysis. By addition of inulinase, inulin is hydrolysed under proportionally consumption of nicotinamide adenine dinucleotide (NADH). NADH was determined by spectrophotometry at 340 nm (Versamax® Micro plate reader, Molecular Devices Corporation, Sunnyvale, CA, USA).

Plasma concentrations of sodium and potassium ions were determined by flame photometry (ILS 943, Instrumentation Laboratory, Lexington, MA, USA), renin was measured by the antibody trapping method of Poulsen and Jorgensen [13], and aldosterone was measured by a commercial radioimmunoassay kit (Coat-A-Count® Aldosterone, Diagnostic Products Corporation, Los Angeles, CA, USA).

### Morphology

Under anaesthesia (fentanyl and fluanisone at doses of 236 μg/kg and 7.5 mg/kg, respectively (Hypnorm®, VetaPharma, Sherburn-in-Elmet, Leeds, UK), midazolam 3.75 mg/kg (Dormicum®, Roche Pharmaceuticals, Basel, Switzerland) administered intraperitoneally, and N_2_O/O_2_ 50/50%) the right kidney was removed, weighed and quick frozen while the left kidney was perfusion-fixed with formaldehyde 4% for 5 min, weighed, divided into slices of 1 mm, and stored in formaldehyde. Subsequently, every second of the 1 mm slices were embedded in paraffin, cut at 3 μm in thickness, and stained with PAS and Masson-Trichrome. Using a light microscope with motorized stage and the CAST-grid analysis software, the volume fractions of tubules and the volume fraction of the interstitial space in the cortex were estimated by point counting in eighty systematically randomly sampled areas from each individual animal [14,15]. In addition, semiquantitative grading (0 to 3) of hyaline vacuolisation in tubules and of vascular hyaline depositions in arterioles was performed.

### Western immunoblotting

Rat renal cortex tissue was homogenized in sucrose/imidazole buffer (0.3 M sucrose, 25 mM imidazole, 1 mM EDTA, pH 7.2. Before use supplemented with protease inhibitors: 0.4 M pefablock, 2.1 mM leupeptin and phosphatase inhibitors: 1 mM Na-ortho-vanadate, 0.2 M NaF and 0.082 μg/μL okadaic acid). Protein was quantified by the Bradford method. For western blotting 10 μg of protein was run in a SDS-PAGE gel. Each sample was added reducing agent (NuPAGE Sample Reducing agent, Invitrogen) and sample buffer (NuPAGE LDS Sample buffer, Invitrogen) and denatured for 5 min at 95°C before running. Proteins were transferred to an activated 0.45-μm pore-size Immobilon-P PVDF-membrane (Millipore) in the blotting system (XCell SureLock Mini-Cell system, Invitrogen). Membranes were blocked with 5% nonfat milk in TBST for one hour before incubation with primary antibody. Primary antibodies used were – α-SMA (ab5694, Abcam, 1:1.000), E-cadherin (610181, BD Biosciences, 1:20.000) and pAkt/PKB (Santa Cruz Biotechnology, 1:2.000). The antigen-antibody complex was visualized by horseradish peroxidase-conjugated secondary antibodies (1:2.000, Dako, Denmark).

### Statistics

Analysis of variance (ANOVA) was applied for comparison of data collected from more than two groups. In case of significance by ANOVA, group-to-group comparisons were done with Dunnett's Multiple Comparison Test. Values are given as mean ± SEM. A p value below 5% was considered statistically significant.

## Results

### Effect of eplerenone on kidney morphological parameters following 12 weeks cyclosporine treatment

**Figure 1:**
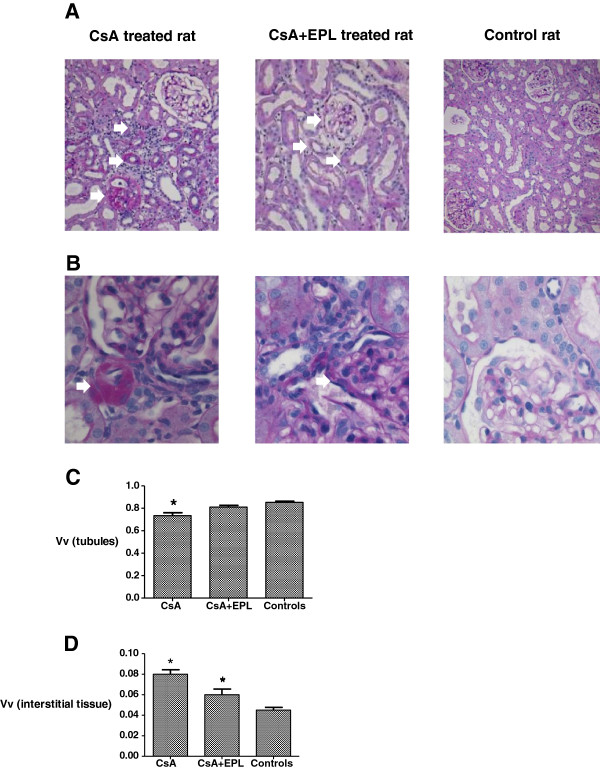
**A. Renal cortex of rats after 12 weeks of treatment with cyclosporine A, cyclosporine A in combination with eplerenone, or controls receiving vehicle only.** In a CsA treated rat, the tissue shows diffuse interstitial inflammation, atrophic tubules, and a sclerotic glomerulus. CsA+EPL treated rat kidney section appears normal and not different from control-vehicle with respect to morphology of the glomerulus, arterioles, and tubules. PAS x100. **B.** Section of renal cortex of rats after 12 weeks of treatment with cyclosporine A, cyclosporine A in combination with eplerenone, or controls receiving vehicle only. In a CsA treated rat there is massive hyaline arteriolar vacuolization whereas after CsA and EPL treatment the hyaline arteriolar vacuolization appears less pronounced and widespread and not very different from a control-vehicle rat. PAS x200. **C.** Stereological quantitation of the volume fraction (Vv) of tubules in renal cortex of rats receiving 12 weeks of treatment with cyclosporine A (n=8) or cyclosporine A in combination with eplerenone (n=9) compared to vehicle- controls (n=11). Values were obtained by a blinded cast-grid analysis counting eighty frames from each individual rat. PAS x100. CsA: Cyclosporine A. EPL: Eplerenone. *) p<0.05 when compared to the control group. **D.** Volume fractions (Vv) of the interstitium in renal cortex of rats receiving 12 weeks of treatment with cyclosporine A (n=8) or cyclosporine A in combination with eplerenone (n=9) compared to controls (n=11). CsA: Cyclosporine A. EPL: Eplerenone. *) p<0.05 when compared to the control group.

Focal tubular atrophy, interstitial inflammation and fibrosis, and more sclerotic glomeruli were observed in response to CsA (Figure 1A). Hyaline tubular casts and tubular dilatation as well as hyaline deposition in arterioles, mainly located close to the glomeruli, were seen in most of the animals treated with CsA; a semiquantitative score of 2 vs. 0.5 (p<0.05) (Figure 1B, Table 1). Stereological quantitation of kidney tissue showed that CsA-treated rat kidneys displayed a significantly lower volume fraction of tubules (Figure 1C) and a significantly expanded volume fraction of renal interstitium compared to controls (Figure 1D). The CsA-induced changes in the relative tubular and interstitial volumes were all significantly attenuated by EPL treatment (Figure 1A, 1C, 1D) (p<0.05), and also the score for hyaline vacuolisation in tubules and of vascular depositions in arterioles were significantly attenuated by EPL treatment (p<0.05) (Figure 1B, Table 1).

**Table 1:** Semiquantitative scoring of hyaline vacuolization in tubules and of vascular depositions in arterioles in renal cortex of rats treated for 12 weeks with cyclosporine A with/without eplerenone

**Variable**	**Units**	**CsA**	**CsA + EPL**	**Controls**
N		8	9	11
Semiquantitative grading	Score 0-3	2* (1–2)	1± (0–2)	0.5± (0–1)

CsA: cyclosporine A. EPL: eplerenone.

Results are shown as median values and range.

*: p<0.05 when compared to the control group.

PASx100.

### Effect of eplerenone on somatic growth

Cyclosporine A (CsA) whole blood concentrations in the CsA-alone treatment group and the combination therapy group were statistically indistinguishable (Table 2). Over the twelve weeks of treatment, the body weight in the CsA group increased significantly less than that of the control rats (p<0.002, Table 2). During combination treatment, the body weight increased at a rate similar to that of the control rats (Table 2). Kidney weight/body weight ratio did not vary significantly between groups (Table 2).

**Table 2:** Animal growth and blood values during 12 weeks of treatment with cyclosporine A with/without eplerenone

**Variables**	**Units**	**CsA**	**CsA + EPL**	**Controls**
N		8	9	11
Increase in BW	grams/week	13.4*± 5.2	22.2± 4.0	23.0± 5.0
Body weight	g	372* ± 14	461 ± 16	486 ± 13
Kidney weight (right)	g	1.4 ± 0.2	1.7 ± 0.2	1.6 ± 0.2
Kidney weight (total)/BW ratio	mg/g	7.8 ± 1.6	7.4 ± 1.2	6.6 ± 0.8
P potassium	mmol/l	5.1 ± 0.9	5.1 ± 0.4	4.8 ± 0.3
P sodium	mmol/l	140.9 ± 4.9	141.7 ± 4.6	143.0 ± 3.7
P renin	mIU/l	7.4 ± 1.9	12.7 ± 3.9^*^	4.0 ± 1.1
P aldosterone	pg/ml	65 ± 14	493 ± 135^*^	57 ± 17
B cyclosporine	ng/ml	3423 ± 549	2346 ± 254	0

B: Whole blood. BW: Body weight. CsA: cyclosporine A. EPL: eplerenone. P: plasma.

Results are shown as mean values +/− SEM.

*: p<0.05 when compared to the control group.

### Effect of eplerenone on kidney function and plasma electrolytes after 12 weeks of cyclosporine treatment

**Figure 2:**
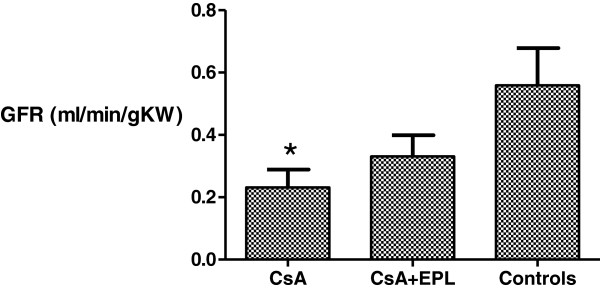
**Glomerular filtration rate as measured by inulin clearance after 12 weeks of treatment with cyclosporine A, cyclosporine A in combination with eplerenone or vehicle-controls.** CsA: Cyclosporine A. EPL: Eplerenone. BW: Body weight. KW: Kidney weight. GFR: Glomerular filtration rate as measured by inulin clearance. *) p<0.05 when compared to the control group.

CsA alone decreased GFR by 56%; concomitant EPL treatment abolished the decrease in GFR (p<0.05, Figure 2). Compared to controls, plasma potassium and plasma sodium did not change during CsA treatment (Table 2). Plasma aldosterone was significantly higher in the EPL-treated group than in controls and in the CsA-only treatment group (p<0.05) (Table 2). Plasma renin was higher in the combination treatment group compared to controls while the animals treated with CsA alone did not have plasma renin levels significantly different from controls (Table 2).

### Effect of eplerenone on blood pressure after 12 weeks cyclosporine treatment

**Figure 3:**
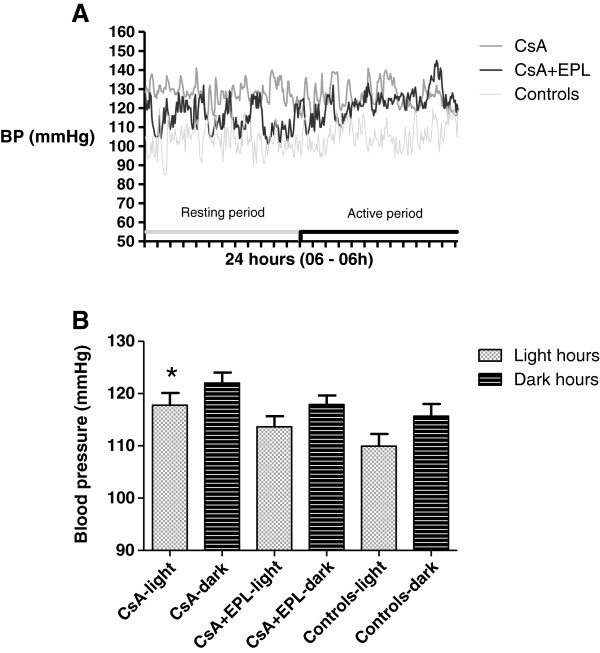
**A. Intra-arterial blood pressure measurement by indwelling chronic catheter during 24 hours after 12 weeks of treatment with cyclosporine A, cyclosporine A in combination with eplerenone or vehicle-control.** Each rat had blood pressure and pulse measured every five minutes during a 96 hours period as the results continually were collected. Mean intra-arterial blood pressure measured continually by chronic indwelling catheters in conscious rats receiving either cyclosporine (CsA), CsA+eplerenone (CsA+EPL), or vehicle (controls). Values showed in 5 minutes intervals for a 24 hours period given as absolute numbers for a single rat from each treatment group. **B.** Intra-arterially mean blood pressure in conscious rats after 12 weeks of treatment with cyclosporine A, cyclosporine A in combination with eplerenone, or controls. Mean intra-arterial blood pressure measured continually by chronic indwelling catheters in conscious rats receiving cyclosporine (CsA), CsA+eplerenone (CsA+EPL), or vehicle (controls). Resting hours: 06:00–18:00; activity hours: 18:00–06:00. Data are means ± SEM. * p<0.05 compared to controls CsA: Cyclosporine A. EPL: Eplerenone. Mean BP: Mean intra-arterial blood pressure.

CsA treatment resulted in significantly higher mean arterial BP daytime as well as night time, when compared to controls (p<0.05, Figure 3B); a typical 24-hour recording is shown in Figure 3A. Interestingly, the combination therapy reduced this effect significantly during daytime; during night hours, the BP tended to be similar to the CsA treated animals (Figure 3B); this tendency is also visible in the 24-hour curves (Figure 3A).

#### Signalling pathways at tissue level

**Figure 4:**
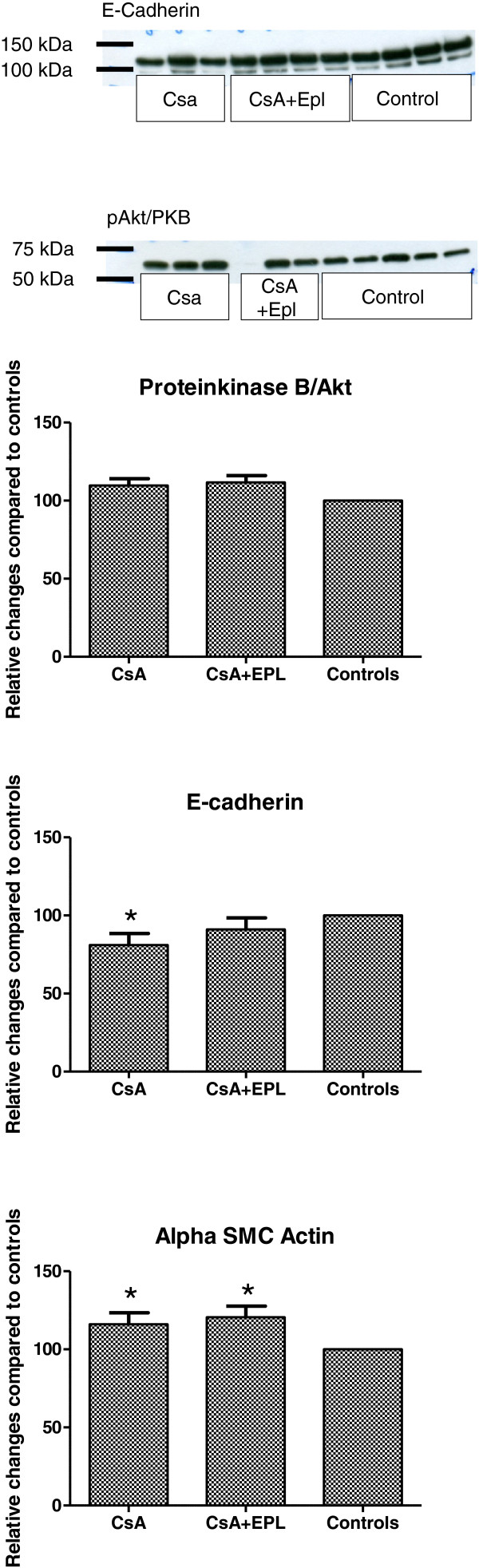
**Analysis of phospho-Akt/PKB (~60 kDa), E-cadherin (~120 kDa), and alpha smooth muscle actin (not shown) protein abundances in renal tissue homogenates from rats receiving 12 weeks of treatment with cyclosporine A, cyclosporine A in combination with eplerenone and vehicle-controls (n=6-8 per condition).** Bar graphs show mean densitometry values from immunoblotting experiments for each condition compared to vehicle control. The same control samples were run on each gel together with CsA-treated and combinated treated rat tissue homogenates. Densitometry values were set to 100% in controls. CsA: Cyclosporine A. EPL: Eplerenone. SMC: Smooth muscle cells. Values are given as relative changes compared to controls. * p<0.05 when compared to the control group.

Western immunoblotting for the epithelial marker E-cadherin in kidney tissue homogenates showed a modest, but significant decrease in abundance in response to CsA (Figure 4), while the mesenchymal cell marker alpha smooth muscle actin was elevated by CsA treatment. EPL did not alter CsA-induced change in alpha smooth muscle actin while the decrease in E-cadherin was attenuated (Figure 4). Abundance of phosphorylated protein kinase B/Akt, a target of activated MR, was not altered by CsA (Figure 4).

## Discussion

The present long-term CsA-nephrotoxicity study shows a significant protective effect of the MR blocker EPL on loss of renal tubules, extracellular matrix expansion, elevated BP, decreased GFR, and impaired somatic growth. Quantitative morphological analysis of renal tissue revealed a preventive effect of EPL on the progressive development of fibrosis and tubular loss relevant for the clinical setting [4-6,8]. In short-term studies, renal function, as measured by GFR, was reduced in CsA-treated animals when compared to controls, but partially preserved in animals in which the CsA treatment was combined with MR-receptor inhibitors [3-8]. The present study indicates that the protection of GFR by EPL is maintained during long-term treatment. Hyperkalemia was not a major challenge in the present or previous studies. A major target for MR is protein kinase B/Akt. The present data did not support an effect of EPL to prevent phosphorylation (activation) of protein kinase B/Akt, as no major changes in renal tissue abundance of phopho-Akt were detected. EPL also did not alter tissue abundance of the mesenchymal cell marker alpha-smooth muscle cell actin, while the CsA-mediated decrease in E-cadherin was attenuated by EPL corroborating a tubular protective effect by EPL. This observation indicates that the apparent interstitial expansion might be secondary to disappearance of tubules rather that primary fibrotic expansion/proliferation. The improved weight gain in EPL-treated rats, compared to the CsA alone treated animals, could be due to direct effects on appetite by EPL or – more likely – by antagonizing the adverse effects of CsA or by reduction of the uraemia and malnutrition arising from the nephrotoxicity. The drop-out rate was low; two cases of fatal infections were seen in the CsA treatment group; that was the case in one of the combination treated rats while no infections were seen in the control rats; this difference was not significant and therefore not a significant confounder.

In absolute terms, mean BP levels in all groups were relatively high which could be related to the social deprivation or the surgical history [16]. However, mean BP in the CsA treatment group was significantly higher than controls in concordance with earlier studies, while the group receiving combination therapy with CsA+EPL had a 24-hour mean BP value that was significantly lower than the CsA treated group. The mean BP in the combination treatment group was not different from that of controls. A subsequent analysis of diurnal variations showed that BP reduction occurred during the resting period (Figure 3A and Figure 3B). During the activity period, rats have an up to ten-fold higher plasma aldosterone level compared to resting day-time periods [17], and data support the notion of diurnal regulation of aldosterone-renal ENaC expression and sodium balance with lower activity during sleep. EPL appears to restore the normal dipping pattern disturbed by CsA. On the other hand, this diurnal fluctuation may account for the variation in EPL efficacy towards BP reduction (night time activation; superimposed stimulation of renin secretion by CsA and further stimulation of aldosterone secretion by EPL [18]). Another explanation of the circadian variation of the EPL effect could be a higher sympathetic tone seen during activity hours at night not significantly influenced by EPL. The beneficial effect of EPL on CsA-induced tissue injury could be accomplished through a direct effect on tubular epithelial target cells or indirectly by the antihypertensive effect. Clinical and experimental animal studies, however, have demonstrated that normalization of BP per se does not prevent deterioration of renal function during CsA-treatment [4,8,19,20]. In accord with a direct effect, calcineurin is co-localized with MR in the distal aldosterone-sensitive segment of the nephron [21].

CsA has been shown to stimulate renin secretion directly [22], and EPL is likely to further stimulate renin indirectly through the increased sodium excretion. In accordance with this we found that plasma renin was higher in the combination treatment group compared to controls (Table 2).

The single, daily dosage of CsA (15 mg/kg/d) was rather high compared to the human clinical setting; however, it was chosen based on results from earlier dose–response studies in this particular rat strain where this dose provided full immunosuppressive effect [1]. Identical whole blood CsA concentrations were obtained in the two groups as in previous nephrotoxicity studies [1,2,23], and at levels similar to peak whole blood concentrations in patients (~2-3 micromol/L). The formula and dosing of EPL was established previously to be effective in rats [10].

We did not use a reduced salt diet as we wanted to analyse a situation resembling as close as possible the clinical setting. In addition, in an earlier short-term study we found a preventive effect of MR-inhibition without using a salt-reduced diet [8].

Clinically, the progressive tissue injury after CsA is a major challenge and although the acute and short-term effects can be prevented to some degree, e.g. by angiotensin converting enzyme inhibitors [4], calcium channel antagonists [19], or angiotensin II receptor blockade [20], these treatments do not improve the long-term clinical outcome. Angiotensin II has been suggested to be responsible for up-regulation of fibrogenic pathways [24], but the observation that MR blockade protects against tissue injury suggests that aldosterone is also involved [25]. The beneficial effect of EPL appears to be a “class effect” since spironolactone has also been shown to prevent CsA nephrotoxicity [3-7] and it appears to be a specific effect not related to BP lowering.

## Conclusion

This long-term experimental animal study of CsA-nephrotoxicity demonstrates a significant protective action of an MR antagonist on tissue injury (reduced renal fibrosis, interstitial expansion, and loss of tubular mass), GFR, BP and the ability to thrive, and suggests that MR antagonists should be tested for their renoprotective actions in clinical settings.

## Abbreviations

ANOVA: Analysis of variance; B: Whole blood; BP: Blood pressure; BW: Body weight; CsA: Cyclosporine A; EPL: Eplerenone; GFR: Glomerular filtration rate; KW: Kidney weight; LD 12:12: 12 hour light and 12 hour dark cycle; MR: Mineralocorticoid receptor; NADH: Nicotinamide adenine dinucleotide; P: Plasma; PAS: Periodic acid Schiff; SEM: Standard error of the mean; SMC: Smooth muscle cells

## Competing interests

The authors declare that they have no competing interests.

## Authors’ contribution

Every one of the authors actively participated in planning the study and interpreting the study results. Everyone also actively contributed in preparing the manuscript. FTN and NM conducted the morphological parts while BLJ performed the Western immunoblotting. All authors read and approved the final manuscript.

## Pre-publication history

The pre-publication history for this paper can be accessed here:

http://www.biomedcentral.com/1471-2369/14/42/prepub
